# Looking to nature for a new concept in antimicrobial treatments: isoflavonoids from *Cytisus striatus* as antibiotic adjuvants against MRSA

**DOI:** 10.1038/s41598-017-03716-7

**Published:** 2017-06-19

**Authors:** Ana Cristina Abreu, Aline Coqueiro, Andi R. Sultan, Nicole Lemmens, Hye Kyong Kim, Robert Verpoorte, Willem J. B. van Wamel, Manuel Simões, Young Hae Choi

**Affiliations:** 1 Natural Products Laboratory, Institute of Biology, Leiden University, Leiden, The Netherlands; 2 LEPABE, Department of Chemical Engineering, Faculty of Engineering, University of Porto, Rua Dr. Roberto Frias, s/n, 4200-465 Porto, Portugal; 3 Department of Medical Microbiology and Infectious Diseases, Erasmus MC, Rotterdam, The Netherlands

## Abstract

The spread of multidrug-resistant *Staphylococcu*s *aureus* strains, including methicillin-resistant *S*. *aureus* (MRSA), has shortened the useful life of anti-staphylococcal drugs enormously. Two approaches can be followed to address this problem: screening various sources for new leads for antibiotics or finding ways to disable the resistance mechanisms to existing antibiotics. Plants are resistant to most microorganisms, but despite extensive efforts to identify metabolites that are responsible for this resistance, no substantial progress has been made. Plants possibly use multiple strategies to deal with microorganisms that evolved over time. For this reason, we searched for plants that could potentiate the effects of known antibiotics. From 29 plant species tested, *Cytisus striatus* clearly showed such an activity and an NMR-based metabolomics study allowed the identification of compounds from the plant extracts that could act as antibiotic adjuvants. Isoflavonoids were found to potentiate the effect of ciprofloxacin and erythromycin against MRSA strains. For the structure-activity relationship (SAR), 22 isoflavonoids were assessed as antibiotic adjuvants. This study reveals a clear synergy between isoflavonoids and the tested antibiotics, showing their great potential for applications in the clinical therapy of infections with antibiotic-resistant microorganisms such as MRSA.

## Introduction

The worldwide spread of multidrug-resistant (MDR) bacteria is increasingly attracting the attention of global surveillance authorities and media, and is undoubtedly rated as a major health threat in the 21st century. Each year over 13 million deaths in the world are attributed to the emergence of new infectious diseases or to the re-emergence of old pathogens with new resistance determinants^1^. This situation affects everyone, independently of age, gender or the country in which they live, and threatens to undermine all recent notable achievements of modern medicine. The fact that only 50 years of use of antibiotics has already led to widespread resistance shows that more sophisticated systems are required to effectively treat infectious diseases.

Natural products of microbial origin have mainly, though not exclusively, been the most important sources of leading antimicrobials for the pharmaceutical industry^2, 3^. Although plants have been studied intensively in the hope of finding novel antibiotics, none of them have lived up to expectations so far. Plants have different levels of defense against microorganisms, including fungi, yeasts and bacteria^4^ – constitutive chemical defense, direct inducible chemical defense (e.g. phytoanticipins) and gene-level inducible chemical defense (e.g. phytoalexins) – thereby producing an array of structurally diverse secondary metabolites. In most cases, studies have led to the isolation of single compounds that were generally not very active in *in vitro* tests^5^, whereas the synergistic interactions between various metabolites could enhance the activity of these otherwise weak antimicrobial agents. Indeed, when isolated, these antimicrobial metabolites exhibit only weak or moderate activity with minimal inhibitory concentrations (MICs) typically in the range of 100–1000 µg/mL, that is, several orders of magnitude above those of typical antibiotics produced by bacteria and fungi (MICs, 0.01–10 µg/mL)^5^, accounting for the absence of plant-derived antimicrobials in clinical applications^6^. A likely explanation for this is that plants, rather than relying on single metabolites, use a combination of strategies and a highly efficient defense system that includes very diverse molecules ranging from proteins to H_2_O_2_ and oxygen radicals, which most likely complement each other, to deal with microbial threats. The defense strategies may thus involve the synergistic activity of two or more compounds, which could act via different mechanisms and/or targets^7^. A great number of plant metabolites have been reported to exhibit these mechanisms involving the inhibition of several MDR efflux pumps for Gram-positive bacteria^4, 8–15^ implicated in bacterial resistance to several antibiotic classes, or to inhibit protein-binding proteins including (PBP)-2a^8, 16, 17^, among others. It is important to bear in mind that millions of years of evolution have resulted in plant defense systems that have proved to be not readily susceptible to microbial resistance mechanisms. It is this capability that triggers the interest in further studies, as there could be a lesson to learn from such an evolutionary successful strategy.

The goal of this study, thus, is to test whether it is possible to take advantage of a plant’s powerful defense system, presumably based on complex synergistic interactions, to potentiate the activity of known antibiotics. Such a complex biological question is perfectly suited to a systems biology approach since a reductionist approach such as the conventional bioassay-guided fractionation cannot reveal interactions between compounds, such as synergy. Indeed, the full understanding of synergy in the biological activities of natural products is a major challenge^18, 19^. In contrast to the classical approach, in which while individually active, albeit weak, antimicrobial compounds may be identified, detecting synergistic interactions would imply many compounds being isolated in sufficient amounts to test them in an almost infinite number of combinations. A metabolomics approach seemed to be more promising. Metabolomics combines the use of a powerful analytical platform for data collection with multivariate data analysis of the collected information. Thus, the metabolites that may be correlated with the synergistic activities are revealed and can then be identified.

*Cytisus striatus* (Fabaceae), commonly known as Portuguese broom, a very abundant shrub in the Iberian Peninsula, was selected from among several other plants^20^ for its strong potentiating activity of ciprofloxacin and erythromycin against one of the most menacing bacterial strains, MRSA, which causes serious, invasive and life-threatening infections worldwide^21^. MRSA strains belonging to CC8, including the common ST239 found in Asia and a Dutch ST8-MRSA, were tested. The starting point for the study was to evaluate the antibiotic-potentiating activity of different types of extracts of this plant with proven antimicrobial activity. All extracts were submitted to ^1^H-NMR-based metabolomics. Supervised multivariate data analysis was then applied to identify the signals related to the high potentiating activity. The next step was to isolate the compounds responsible for these signals by NMR-based metabolomics guided fractionation, identify them and test various combinations for a synergistic antimicrobial potentiating activity on MRSA strains.

Fabaceae plants have been studied extensively for their phytoalexins, antimicrobial isoflavonoids that are produced after infection^22^. Isoflavonoids themselves are well known for their therapeutic properties, as they have anti-inflammatory, estrogenic, anti-estrogenic, anticancer, antibiotic and radical scavenging activities^23–26^. Their antimicrobial activity has been reported to be low or moderate in most cases^18, 27, 28^, but they are seemingly involved in the inducible defense response of plants in this family. However, their mechanism of action remains largely unexplained. Similarly, the potentiating activity on ciprofloxacin against *S*. *aureus* ^19, 29–31^, described for some isoflavonoids, is not yet understood. The study showed that isoflavonoids were involved in the potentiating effect in *C*. *striatus*. Based on these results, a further 22 isoflavonoids from different sources were evaluated for their antibacterial and synergistic effects in combination with ciprofloxacin and erythromycin against *S*. *aureus* strains.

## Results

### Antibacterial and antibiotic synergistic activities of *C*. *striatus* leaf, flower and twig methanolic extracts

Seven *S*. *aureus* strains were included in the experiments. Testing different bacterial strains should show whether the mode of action of the plant extract and its interaction with the antibiotic depends on the resistance profile of the strains. The characterization of the strains is described in Table 1. Two ST239-type MRSA strains harboring staphylococcal cassette chromosome *mec* (SCC*mec*) were chosen: RWW337 (from Malaysia) and M116 (from Indonesia). ST239-type MRSA strains are probably the most predominant clone of MRSA causing hospital-acquired infections^33^. ST8 CA-MRSA with SCC*mec*IV (USA300) has been predominant, causing infections outside the health-care environment in the United States^34^. In this study, we investigated one Dutch ST8-MRSA (RWW50) carrying the SCC*mec* element. One ST20 clinical *S*. *aureus* strain (M82), also from Indonesia, was included as well. The Laboratory strains *S*. *aureus* RN6390 (a natural respiratory epithelial cell line that is a *sigB*-deficient mutant) and CECT 976 (from the Spanish collection), already used as a model microorganism for antimicrobial tests with phytochemical compounds^35, 36^, were included in the experiments. *S*. *aureus* SA1199B, derived from a methicillin-susceptible *S*. *aureus* (MSSA) bloodstream isolate from a patient with endocarditis, was used as a control strain since it overexpresses the NorA MDR efflux pump. The MICs of ciprofloxacin and erythromycin were determined for each strain by microdilution techniques according to the Clinical and Laboratory Standards Institute (CLSI) guidelines^32^ and also showed in Table 1. The MIC of the ethyl acetate fractions of the methanolic extracts of *C*. *striatus* leaves, flowers and twigs were also determined against the seven *S*. *aureus* strains. None of the extracts exhibited a MIC below 1 mg/mL against the *S*. *aureus* strains (Supplementary Table 1).

**Table 1:** Characterization of *Staphylococcus aureus* strains.

*S*. *aureus*	CIP	ERY	Origin	Relevant characteristics
CECT 976	1.0 (S)	0.25 (S)	Laboratory strain	From Spanish Type Culture Collection; equivalent to ATCC 13565; no antibiotic resistance
RN6390	0.25 (S)	0.5 (S)	Laboratory strain	ST8; no antibiotic resistance
SA1199B	4 (R)	n.p.	Laboratory strain	NorA-overproducing derivative of a clinical isolate, SA-1199, also has A116E GrlA substitution, MSSA
M82	0.25 (S)	0.5 (S)	Indonesia (CI)	ST20-MSSA
M116	32 (R)	32 (R)	Indonesia (CI)	ST239-MRSA
RWW337	16 (R)	>10 000 (R)	Malaysia (CI)	ST239-MRSA
RWW50	2 (I)	64 (R)	Laboratory strain (collection MMIZ Erasmus MC)	ST8-MRSA

Minimal inhibitory concentrations (MICs) of ciprofloxacin (CIP) and erythromycin (ERY) were determined for each strain (µg/mL) according to CLSI guidelines^32^, which were classified as resistant (R), intermediate (I) or susceptible (S) to each antibiotic. Origin and genetic profiles of the strains are described.

n.p. not performed.

CI – clinical isolate.

The methanolic extracts of leaves, flowers and twigs of *C*. *striatus* (EtOAc fractions with a concentration of 0.5 mg/mL) were assessed for their potentiating effect of ciprofloxacin and erythromycin activity against the seven *S*. *aureus* strains, using the disk diffusion method (Table 2). The different parts of the plant showed different antibiotic-potentiating activities. The leaf extract showed the best potentiating effect on both ciprofloxacin and erythromycin against several strains. Potentiation was also obtained when combinations were tested with the checkerboard assay (Supplementary Table 1). The flower extract showed additive and potentiating effects on the antibiotics only against *S*. *aureus* strains CECT 976 and SA1199B, respectively. The twig extract showed no antibiotic-potentiating activity against the tested seven *S*. *aureus* strains. The inhibition zone diameter (IZD) of each combination was never lower than that produced by each antibiotic alone (*P* > 0.05), proving that there was no antagonism detected in the combinations.

**Table 2:** Antibiotic-potentiating activity of *Cytisus striatus* extracts on different *Staphylococcus aureus* strains.

Increased IZD (IZD_c_ – IZD_a_) (mm)						
*S*. *aureus* strains	Leaf		Flower		Twig	
	CIP	ERY	CIP	ERY	CIP	ERY
CECT 976	6.3 ± 1.0 (P)	9.1 ± 0.6 (P)	5.4 ± 1.2 (A)	4.5 ± 1.0 (A)	—	—
M116	7.0 ± 1.0 (P)	10.0 ± 0.0 (P)	—	—	—	—
RWW337	8.3 ± 0.6 (P)	—	—	—	—	—
RWW50	7.0 ± 0.0 (P)	5.0 ± 1.0 (A)	—	—	—	—
M82	4.0 ± 0.0 (A)	4.5 ± 0.0 (A)	—	—	—	—
RN6390	—	5.0 ± 0.3 (A)	—	—	—	—
SA1199B	14.0 ± 2.5 (P)	n.p.	17.3 ± 0.3 (P)	n.p.	—	n.p.

The activity is expressed as the increase in the inhibition zone diameters (IZDs, mm) caused by ciprofloxacin (CIP) or erythromycin (ERY) when a plant extract is present in the Mueller-Hinton agar medium. The methanolic extracts of *C*. *striatus* leaves, flowers and twigs were applied at 0.5 mg/mL. Inhibition zones obtained with the combinations (IZD_c_) over antibiotic-single activity (IZD_a_) are given and the combinations are classified as: (—) indifferent (IZD_c_ – IZD_a_ < 4 mm); (A) additive (4 ≤ IZD_c_ – IZD_a_ < 6 mm); and (P) potentiation (IZD_c_ – IZD_a_ ≥ 6 mm)^20, 35, 36^. Data are means and SD from at least three independent experiments.

n.p. not performed.

### ^1^H-NMR measurement of the extracts of different parts of C. *striatus*

**Figure 1:**
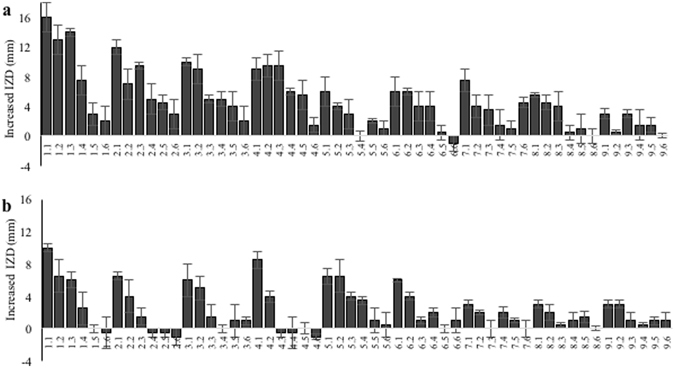
Potentiating effects of ciprofloxacin and erythromycin obtained when combined with the different extracts of *Cytisus striatus* against *Staphylococcus aureus* CECT 976. The activity is expressed as the increase in the inhibition zone diameter (IZD, mm) caused by ciprofloxacin (**a**) and erythromycin (**b**) in the presence of the extracts of *C*. *striatus* dissolved in Mueller-Hinton agar medium. Bars represent means and SD from at least three independent experiments. The sample parameters (*x*,*y*) are shown in Supplementary Table 2.

^1^H-NMR was applied to obtain metabolic profiles of all samples^37^. Visual inspection of the ^1^H-NMR spectra of the EtOAc fractions of leaf, flower and twig extracts revealed some differences in their chemical profiles (Supplementary Fig. 1), especially in the phenolic region, which could be responsible for differences in the antibiotic-potentiating activity: leaves (most active) > flower > twig (nonactive). Different extracts were prepared to enable the use of multivariate data analysis to identify the markers of activity (Supplementary Fig. 2). A high chemical variation is essential to significantly correlate activity and chemical data. Therefore, leaves or flowers (active parts) were mixed with twigs (nonactive parts) in various ratios in order to obtain this variation. These mixtures were extracted using different solvents, temperature and pressure conditions (Supplementary Table 2). The resulting 54 extracts (EtOAc fractions) were assayed for their potentiation of the activity of ciprofloxacin and erythromycin against *S*. *aureus* CECT 976 using the disk diffusion method (Fig. 1). In general, extracts obtained with 100% methanol showed a higher potentiating activity than those prepared with 75- and 50% MeOH in water (*P* < 0.05); temperature and pressure did not significantly affect the activity (*P* < 0.05).

**Table 3:** Main metabolites detected in active fraction of *Cytisus striatus* leaves.

	Chemical formula	^1^H-NMR (CD_3_OD-d4; δ (ppm))
Apigenin	C_15_H_9_O_5_	6.19 (H6, d, *J* = 1.9), 6.43 (H8, d, *J* = 1.9), 6.57 (H3, s), 6.92 (H3′/H5′, d, *J* = 8.8), 7.84 (H2′/H6′, d, *J* = 8.8)
Chrysin	C_15_H_10_O_4_	6.18 (H6, d, *J* = 1.9), 6.42 (H8, d, *J* = 1.9), 6.70 (H3, s), 7.56 (H3′/H4′/H5′, m); 7.97 (H2′/H6′, d, *J* = 7.8)
Luteolin	C_15_H_9_O_6_	6.19 (H6, d, *J* = 1.9), 6.42 (H8, d, *J* = 1.9), 6.53 (H3, s), 6.89 (H5′, d, *J* = 8.4), 7.37 (H6′, dd, *J* = 8.4, 1.9), 7.37 (H2′, d, *J* = 1.9)
Daidzein	C_15_H_10_O_4_	6.83 (H3′/H5′, d, *J* = 8.4), 6.84 (H8, d, *J* = 1.8), 6.92 (H6, dd, *J* = 9.0, 2.4), 7.36 (H2′/H6′, d, *J* = 8.4), 8.04 (H5, d, *J* = 8.4), 8.12 (H2, s)
3′-Hydroxydaidzein	C_15_H_9_O_5_	6.79 (H8, d, *J* = 1.8), 6.82 (H5′/H6′, m), 6.90 (H6, dd, *J* = 9.0, 2.4),7.00 (H2′, d, *J* = 1.8), 8.02 (H5, d, *J* = 8.4), 8.08 (H2, s),
Genistein	C_15_H_9_O_5_	6.20 (H6, d, *J* = 2.1), 6.32 (H8, d, *J* = 2.1), 6.84 (H3′/H5′, d, *J* = 8.4), 7.36 (H2′/H6′, d, *J* = 8.4), 8.04 (H2, s)
2′-Hydroxygenistein	C_15_H_10_O_6_	6.15 (H6, d, *J* = 1.8), 6.26 (H8, d, *J* = 1.8), 6.37 (H5′, dd, *J* = 7.8, 2.4), 6.39 (3′H, d, *J* = 1.8), 7.03 (H6′, d, *J* = 8.4), 7.95 (H2, s)

^1^H-NMR data are measured in ppm and coupling constants (*J*) in Hertz.

**Figure 2:**
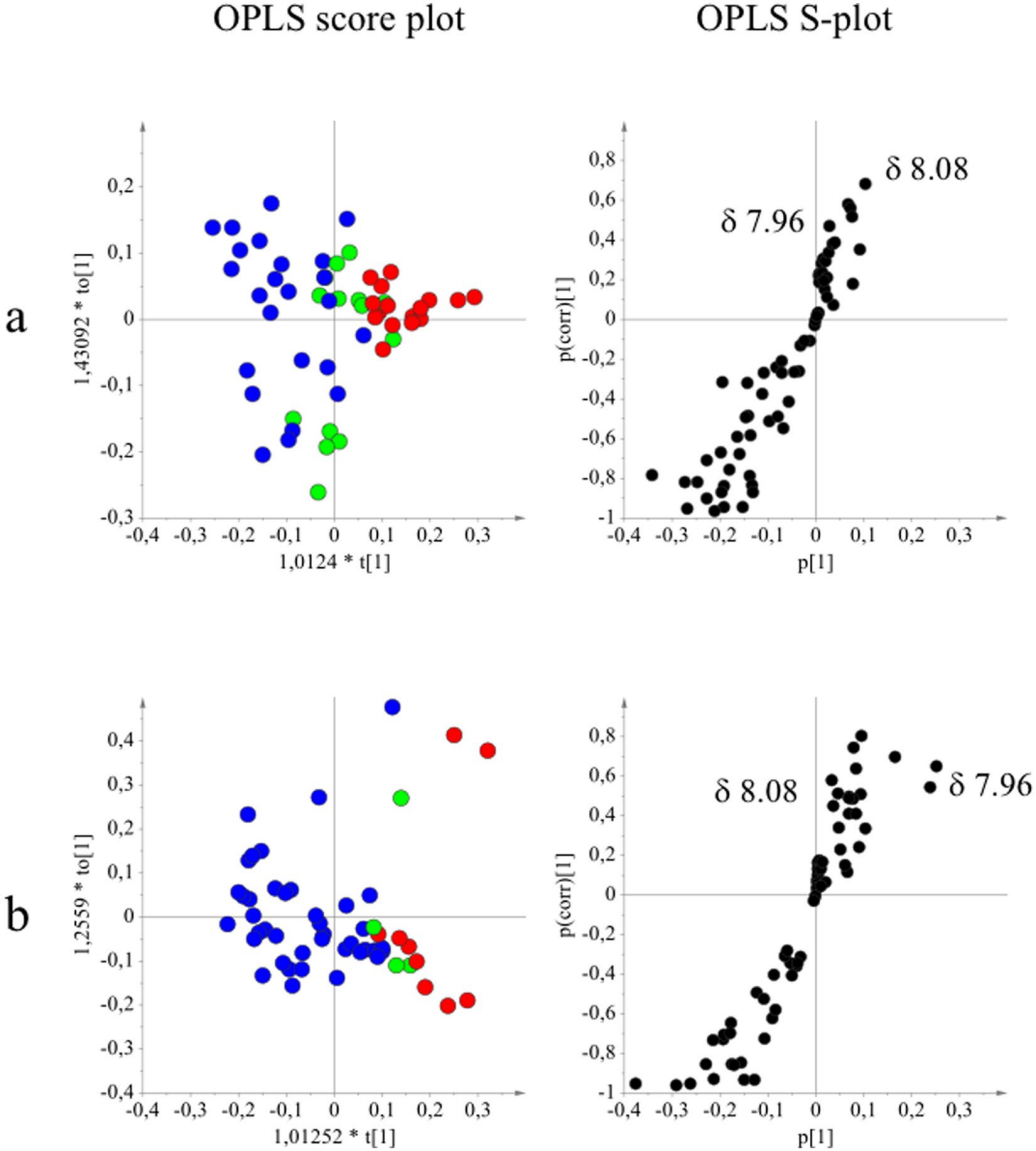
Orthogonal partial least squares modeling applied to ^1^H-NMR data and potentiating activity of *Cytisus striatus* samples (Fig. 2). OPLS score- and S-plots obtained from the potentiating activity (1: indifferent , 2: additive  and 3: potentiation effect ) and ^1^H-NMR data in the range of the region between *δ* 6.00 and 8.60 of the different classes of potentiating activity of extracts of *C*. *striatus* on ciprofloxacin (**a**) and erythromycin (**b**). The sample preparation and extraction conditions were performed as shown in Supplementary Table 2.

The ^1^H-NMR data obtained for the 54 samples were correlated with potentiating activity data by orthogonal partial least squares (OPLS) modeling to investigate the grouping of the samples according to the potentiating activity of ciprofloxacin (Fig. 2a) and erythromycin (Fig. 2b). The aromatic region (δ 6.0–8.6) was considered since phenolic compounds were expected to have a higher therapeutic potential. Both OPLS score plots of ciprofloxacin and erythromycin showed that the phenolic ingredients correlated well with the activity, which was confirmed by a permutation test. An S-plot was used to identify the metabolites that contributed to the activity. The signals in the range of δ 7.9–8.2, e.g. H-2 of isoflavonoids or H-2′ and H-6′ of flavonoids with a 4′-monohydroxy group in the B-ring, were found to strongly correlate with the potentiating activity. To confirm the chemical structures associated with this activity, the *C*. *striatus* leaf methanolic extracts were separated using column chromatography with silica gel and then with Sephadex LH-20 yielding two fractions, B5 and B6, that exhibited distinct activities. The B5 fraction potentiated the activity of ciprofloxacin and erythromycin (similarly to *C*. *striatus* extract) and B6 showed antibacterial activity against *S*. *aureus* CECT 976. The B6 fraction was identified as luteolin. Semi-preparative RP-HPLC of the B5 fraction enabled the separation of several compounds that were identified by ^1^H-NMR as apigenin, chrysin, daidzein, genistein, 2′-hydroxygenistein and 3′-hydroxydaidzein. All compounds were identified using the in-house library of NMR data of common metabolites. When necessary, 2D-NMR techniques were used to confirm the identification (*J*-resolved, ^1^H-^1^H COSY, ^1^H-^13^C HMBC and ^1^H-^13^C HSQC) (Table 3).

### Antibacterial evaluation of the single compounds isolated from *C*. *striatus* leaves

The MICs of luteolin, apigenin, chrysin, genistein and daidzein against *S*. *aureus* strains were determined (Table 4). Luteolin was the only compound that showed antibacterial activity against *S*. *aureus* strains with a very mild MIC of between 30 and 120 µg/mL. The potentiation effect of the five isolated compounds on the activity of ciprofloxacin and erythromycin was tested using the checkerboard assay, and fractional inhibitory concentration index (FICI) values were calculated. The minimal concentration of compounds that exhibited antibiotic potentiation is shown for each strain. Synergy (for luteolin) or potentiation (for the nonantibacterial phytochemicals) was considered to be present for values of FICI ≤ 0.5. No synergistic effects were obtained with luteolin when combined with ciprofloxacin or erythromycin (FICI > 0.5). The MIC of ciprofloxacin against RWW337 and M116 was reduced 4- and 8-fold by apigenin (15–60 µg/mL) and its isoflavone analogue genistein (30–60 µg/mL), respectively. Genistein also reduced the MIC of ciprofloxacin against SA1199B 8-fold (reversing the resistance of this strain), but not apigenin. Daidzein (60 µg/mL) only potentiated ciprofloxacin against SA1199B (4-fold reduction). Genistein produced a 4-fold reduction of the MIC of erythromycin against RWW337, M116 and RWW50 MRSA strains. No potentiating effect was obtained with chrysin. No potentiation effect was observed with any of the compounds against the susceptible strains CECT 976, M82 and RN6390, except for genistein against CECT 976 (4-fold reduction of the MIC of ciprofloxacin and erythromycin).

**Table 4:** Antibiotic-potentiating activity of the isolated compounds.

		CIP					ERY				
		Lut	Apig	Chry	Gen	Daid	Lut	Apig	Chry	Gen	Daid
M116	MIC_a_	30	>120	>120	>120	>120	30	>120	>120	>120	>120
	MIC_b_	—	30	—	60	15	3.5	30	—	30	—
	R	—	4	—	4	2	2	4	—	4	—
	FICI	I	**≤0**.**38** (**P**)	I	**≤0**.**50** (**P**)	>0.5 (I)	0.63 (I)	**≤0**.**38** (**P**)	I	**≤0**.**38** (**P**)	I
RWW337	MIC_a_	30	>120	>120	>120	>120	30	>120	>120	>120	>120
	MIC_b_	—	60	—	60	—	—	30	—	60	—
	R	—	8	—	8	—	—	≥2	—	≥4	—
	FICI	I	**≤0**.**38** (**P**)	I	**≤0**.**38** (**P**)	I	I*	(*)	I*	**≤0**.**50** (**P**)*	I*
RWW50	MIC_a_	60	>120	>120	>120	>120	30	>120	>120	>120	>120
	MIC_b_	—	15	—	30	—	—	—	—	60	—
	R	—	2	—	2	—	—	—	—	4	—
	FICI	I	>0.5 (I)	I	>0.5 (I)	I	I	I	I	**≤0.50 (P)**	I
M82	MIC_a_	120	>120	>120	>120	>120	60	>120	>120	>120	>120
	MIC_b_	60	30	—	30	—	60	—	—	30	—
	R	4	2	—	2	—	4	—	—	2	—
	FICI	0.75 (I)	>0.5 (I)	I	>0.5 (I)	I	0.75 (I)	I	I	>0.5 (I)	I
RN6390	MIC_a_	120	>120	>120	>120	>120	120	>120	>120	>120	>120
	MIC_b_	—	—	—	—	—	—	—	—	—	—
	R	—	—	—	—	—	—	—	—	—	—
	FICI	I	I	I	I	I	I	I	I	I	I
CECT 976	MIC_a_	120	>120	>120	>120	>120	120	>120	>120	>120	>120
	MIC_b_	—	—	—	30	30	—	—	—	30	—
	R	—	—	—	4	2	—	—	—	4	—
	FICI	I	I	I	**≤0**.**38** (**P**)	>0.5 (I)	I	I	I	**≤0**.**38** (**P**)	I
SA1199B	MIC_a_	120	>120	>120	>120	>120	n.p.				
	MIC_b_	—	10	—	60	60					
	R	—	2	—	8	4					
	FICI	I	>0.5 (I)	I	**≤0**.**38** (**P**)	**≤0**.**25** (**P**)					

Minimal inhibitory concentrations (MICs, µg/mL) of the compound isolated from *Cytisus striatus* against clinical *Staphylococcus aureus*, RN6390 and SA1199B when applied alone (MIC_a_) and in combination (MIC_b_) with ciprofloxacin or erythromycin. The MICs of antibiotics are shown in Table 1. Fold reductions of antibiotic MICs in the presence of each phytochemical are also represented (R) as well as Fractional Inhibitory Concentration Index (FICI) values. When FICI ≤ 0.5 (in bold), if the phytochemical has a determinable MIC value itself, the effect is considered as synergy (S); if the phytochemical has no detectable MIC, the effect is called potentiation (P). If FICI > 0.5, the interaction is considered indifferent (I). Erythromycin was not tested against SA1199B. The values presented are the averages of at least three independent assays.

= no decrease or increase in the MIC was observed; n.p. not performed; *no MIC was detected for erythromycin alone against RWW337, but when combined with apigenin and genistein, MIC for erythromycin was found to be at least ½ (not conclusive) and ¼ of the maximal concentration tested, respectively. FICI = FIC(A) + FIC(B), with FIC(A) being the ratio between the MIC of drug A in combination and the MIC of drug A alone and FIC(B) the ratio of the MIC of drug B in combination and the MIC of drug B alone.

Further tests are needed to determine whether the activity of the main antibacterial compound produced by the plant, luteolin, against *S*. *aureus* strains could also be increased in the presence of other metabolites. However, detecting the high number of possible dual and multiple interactions of the compounds as well as their optimal doses is quite challenging. To assess and predict real interactions of the compounds, it would be valuable to test the effects of such combinations on plant pathogens. This would allow a better insight into the defense system of this plant.

### Structure activity relationship of isoflavonoids as antibiotic potentiators

To analyze the structure-activity relationship (SAR), 22 different isoflavonoids (Table 5) were analyzed for their antibacterial and antibiotic-potentiating properties. Table 6 shows the MICs obtained for the active isoflavonoids alone and combined with ciprofloxacin and erythromycin. Only three of these isoflavonoids showed antibacterial activity below 120 µg/mL: neobavaisoflavone, corylifol A and orobol. Synergy was observed with neobavaisoflavone combined with ciprofloxacin against SA1199B (FICI of 0.5) and corylifol A combined with both ciprofloxacin and erythromycin against RWW337 (FICIs of 0.38). Biochanin A (15–60 µg/mL) and tectorigenin (60–120 µg/mL) showed a similar activity to genistein (30–60 µg/mL) when combined with ciprofloxacin and erythromycin against all cell lines except for the susceptible strains CECT 976, M82 and RN6390, for which no activity was found. Additionally, calycosin, irisflorentin, irigenin and daidzein (60 µg/mL) were also able to reduce the MIC of ciprofloxacin 4-fold against the NorA overexpressing strain SA1199B.

**Table 5:** Structures of flavonoids and isoflavonoids found and/or tested in this study.

Flavonoid structure				Isoflavonoid structure					
	
Compounds:	Substituents at carbon position:								
	5	6	7		8	2′	3′	4′	5′
Flavonoids									
Apigenin*	OH	—	OH		—	—	—	OH	—
Chrysin*	OH	—	OH		—	—	—	—	—
Luteolin*	OH	—	OH		—	—	OH	OH	—
Isoflavonoids									
Daidzein*	—	—	OH		—	—	—	OH	—
3′-Hydroxydaidzein*	—	—	OH		—	—	OH	OH	—
Daidzin	—	—	O—glc		—	—	—	OH	—
Corylifol A**	—	—	OH		—	—	—	OH	R_1_
Neobavaisoflavone***	—	—	OH		—	—	R_2_	OH	—
Genistein*	OH	—	OH		—	—	—	OH	—
2′-Hydroxygenistein*	OH		OH		—	OH	—	OH	—
Genistin	OH	—	O—glc		—	—	—	OH	—
Orobol	OH		OH				OH	OH	
Calycosin	—	—	OH		—	—	OH	OCH_3_	—
Calycosin-7-O-β-D-glucoside	—	—	O—glc		—	—	OH	OCH_3_	—
Formononetin	—	—	OH		—	—	—	OCH_3_	—
Ononin	—	—	O-glc		—	—	—	OCH_3_	—
Biochanin A	OH	—	OH		—	—	—	OCH_3_	—
Tectorigenin	OH	OCH_3_	OH		—	—	—	OH	—
Tectoridin	OH	OCH_3_	O-glc		—	—	—	OH	—
Glycitein	—	OCH3	OH		—	—	—	OH	—
Glycitin	—	OCH_3_	O-glc		—	—	—	OH	—
Irigenin	OH	OCH_3_	OH		—	—	OH	OCH_3_	OCH_3_
Iridin	OH	OCH_3_	O-glc		—	—	OH	OCH_3_	OCH_3_
Puerarin	—	—	OH		glc	—	—	OH	—
3′-Hydroxypuerarin	—	—	OH		glc	—	OH	OH	—
3′-Methoxypuerarin	—	—	OH		glc	—	OCH_3_	OH	—
Irisflorentin****	OCH_3_	R_3_	R_3_		—	OCH_3_	OCH_3_	OCH_3_	—
		

Compounds with (*) were isolated from *C*. *striatus* leaves.

**Table 6:** Antibiotic-potentiating active isoflavonoids.

CIP												
		Neob	CorA	Orob	Gen	Tect	BiochA	Calyc	Irig	Gly	Daid	Irisfl
M116	MIC_a_	20	2.5	120	>120	>120	>120	>120	>120	>120	>120	>120
	MIC_b_	5	—	60	60	60	30	—	—	—	15	—
	R	2	—	8	4	2	4	—	—	—	2	—
	FICI	0.75 (I)	I	0.63 (I)	**≤0**.**50** (**P**)	>0.5 (I)	**≤0**.**38** (**P**)	I	I	I	>0.5 (I)	I
RWW337	MIC_a_	20	20	120	>120	>120	>120	>120	>120	>120	>120	>120
	MIC_b_	5	5	60	60	60	30	30	60	—	—	—
	R	2	8	4	8	4	4	2	2	—	—	—
	FICI	0.75 (I)	**0.38 (S)**	0.63 (I)	**≤0**.**38** (**P**)	**≤0**.**50** (**P**)	**≤0**.**38** (**P**)	>0.5 (I)	>0.5 (I)	I	I	I
RWW50	MIC_a_	20	2.5	120	>120	>120	>120	>120	>120	>120	>120	>120
	MIC_b_	10	—	—	30	60	15	30	30	—	—	—
	R	4	—	—	2	2	2	2	2	—	—	—
	FICI	0.75 (I)	I	I	>0.5 (I)	>0.5 (I)	>0.5 (I)	>0.5 (I)	>0.5 (I)	I	I	I
M82	MIC_a_	20	2.5	60	>120	>120	>120	>120	>120	>120	>120	>120
	MIC_b_	10	1.25	—	30	30	30	—	60	—	—	—
	R	4	2	—	2	2	2	—	2	—	—	—
	FICI	0.75 (I)	1 (I)	I	>0.5 (I)	>0.5 (I)	>0.5 (I)	I	I	I	I	I
RN6390	MIC_a_	20	2.5	60	>120	>120	>120	>120	>120	>120	>120	>120
	MIC_b_	10	—	—	—	—	—	—	—	-	—	—
	R	4	—	—	—	—	—	—	—	—	—	—
	FICI	0.75 (I)	I	I	I	I	I	I	I	I	I	I
CECT 976	MIC_a_	20	2.5	60	>120	>120	>120	>120	>120	>120	>120	>120
	MIC_b_	10	—	—	30	60	60	60	60	—	30	60
	R	2	—	—	4	2	2	2	2	—	2	2
	FICI	1.0 (I)	I	I	**≤0**.**38** (**P**)	>0.5 (I)	>0.5 (I)	>0.5 (I)	>0.5 (I)	I	>0.5 (I)	>0.5 (I)
SA1199B	MIC_a_	20	0.06	60	>120	>120	>120	>120	>120	>120	>120	>120
	MIC_b_	5	—	15	60	60	30	60	60	60	60	60
	R	4	—	2	8	4	8	4	4	2	4	4
	FICI	**0.50 (S)**	I	>0.5 (I)	**≤0**.**38** (**P**)	**≤0**.**50** (**P**)	**≤0**.**38** (**P**)	**≤0**.**50** (**P**)	**≤0**.**50** (**P**)	>0.5 (I)	**≤0**.**25** (**P**)	**≤0**.**50** (**P**)
**ERY**												
M116	MIC_a_	20	2.5	120	>120	>120	>120	>120	>120	>120	>120	>120
	MIC_b_	—	1.25	—	30	60	15	30	60	—	—	—
	R	—	2	—	4	4	4	2	2	—	—	—
	FICI	I	1 (I)	I	**≤0**.**38** (**P**)	**≤0**.**38** (**P**)	**≤0**.**28** (**P**)	>0.5 (I)	>0.5 (I)	I	I	I
RWW337	MIC_a_	20	20	120	240	>240	>240	>240	>240	>120	>120	>120
	MIC_b_	—	5	60	60	120	60	—	—	—	—	—
	R	—	≥8	≥4	**≥4**	≥2	**≥4**	—	—	—	—	—
	FICI	I*	**≤0**.**38** (**S**)*	>0.50 (I)*	**≤0**.**50** (**P**)*	(*)	**≤0**.**50** (**P**)*	I*	I*	I	I	I
RWW50	MIC_a_	20	2.5	120	>120	>120	>120	>120	>120	>120	>120	>120
	MIC_b_	10	—	—	60	60	60	30	60	—	—	—
	R	8	—	—	4	4	4	2	2	—	—	—
	FICI	0.63 (I)	I*	I*	**≤0**.**50** (**P**)	**≤0**.**50** (**P**)	**≤0**.**50** (**P**)	>0.5 (I)	>0.5 (I)	I	I	I
M82	MIC_a_	20	2.5	60	>120	>120	>120	>120	>120	>120	>120	>120
	MIC_b_	5	1.25	—	30	60	30	120	60	—	—	—
	R	2	2	—	2	2	2	2	2	—	—	—
	FICI	0.75 (I)	1 (I)	I	≥0.56 (I)	≥0.63 (I)	≥0.56 (I)	>0.5 (I)	>0.5 (I)	I	I	I
RN6390	MIC_a_	20	2.5	60	>120	>120	>120	>120	>120	>120	>120	>120
	MIC_b_	10	—	—	—	—	—	—	—	—	—	—
	R	4	—	—	—	—	—	—	—	—	—	—
	FICI	0.75 (I)	I	I	I	I	I	I	I	I	I	I
CECT 976	MIC_a_	20	2.5	60	>120	>120	>120	>120	>120	>120	>120	>120
	MIC_b_	—	1.25	—	30	60	—	60	60	—	—	—
	R	—	2	—	4	2	—	2	2	—	—	—
	FICI	I	1 (I)	I	**≤0**.**38** (**P**)	>0.5 (I)	I	>0.5 (I)	>0.5 (I)	I	I	I

Minimal inhibitory concentrations (MICs, µg/mL) of the isoflavonoids against clinical *S*. *aureus*, RN6390 and SA1199B when applied alone (MIC_a_) and in combination (MIC_b_) with ciprofloxacin or erythromycin. The MICs of antibiotics are shown in Table 1. Fold reductions of antibiotic MICs in the presence of each isoflavonoid are also represented (R) as well as Fractional Inhibitory Concentration Index (FICI) values. When FICI ≤ 0.5 (in bold), if the isoflavonoid has a determinable MIC value, the effect is considered as synergy (S); if the isoflavonoid has no detectable MIC, the effect is called potentiation (P). If FICI > 0.5, the interaction is considered indifferent (I). Nonactive isoflavonoids are not included. ERY was not tested against SA1199B. The values presented are the averages of three independent assays.

= no decrease or increase in the MIC was observed; n.p. not performed; *no MIC was detected for erythromycin alone against RWW337, but when combined with genistein and biochanin A, erythromycin MIC was found to be at least ¼ of the maximal concentration tested, and at least ½ for tectorigenin. FICI = FIC(A) + FIC(B), with FIC(A) being the ratio between the MIC of drug A in combination and the MIC of drug A alone and FIC(B) the ratio of the MIC of drug B in combination and the MIC of drug B alone.

### Effect of isoflavonoids on ethidium bromide (EtBr) accumulation

Resistance against antibiotics in *S*. *aureus* is, among others, related to MDR efflux pumps such as NorA (MFS family, for which several fluoroquinolones, monocationic dyes and disinfectants are substrates), which have been studied extensively. The potential of isoflavonoids to inhibit efflux pumps was assessed fluorometrically with an efflux accumulation assay of EtBr at ½ MIC to avoid compromising the cellular viability (MIC determination is shown in Supplementary Experiment 1). It has been observed that the accumulation of EtBr increased in bacterial cells in the presence of an efflux pump inhibitor (EPI), which inhibits the EtBr efflux activity of Gram-positive bacteria^38, 39^.

**Figure 3:**
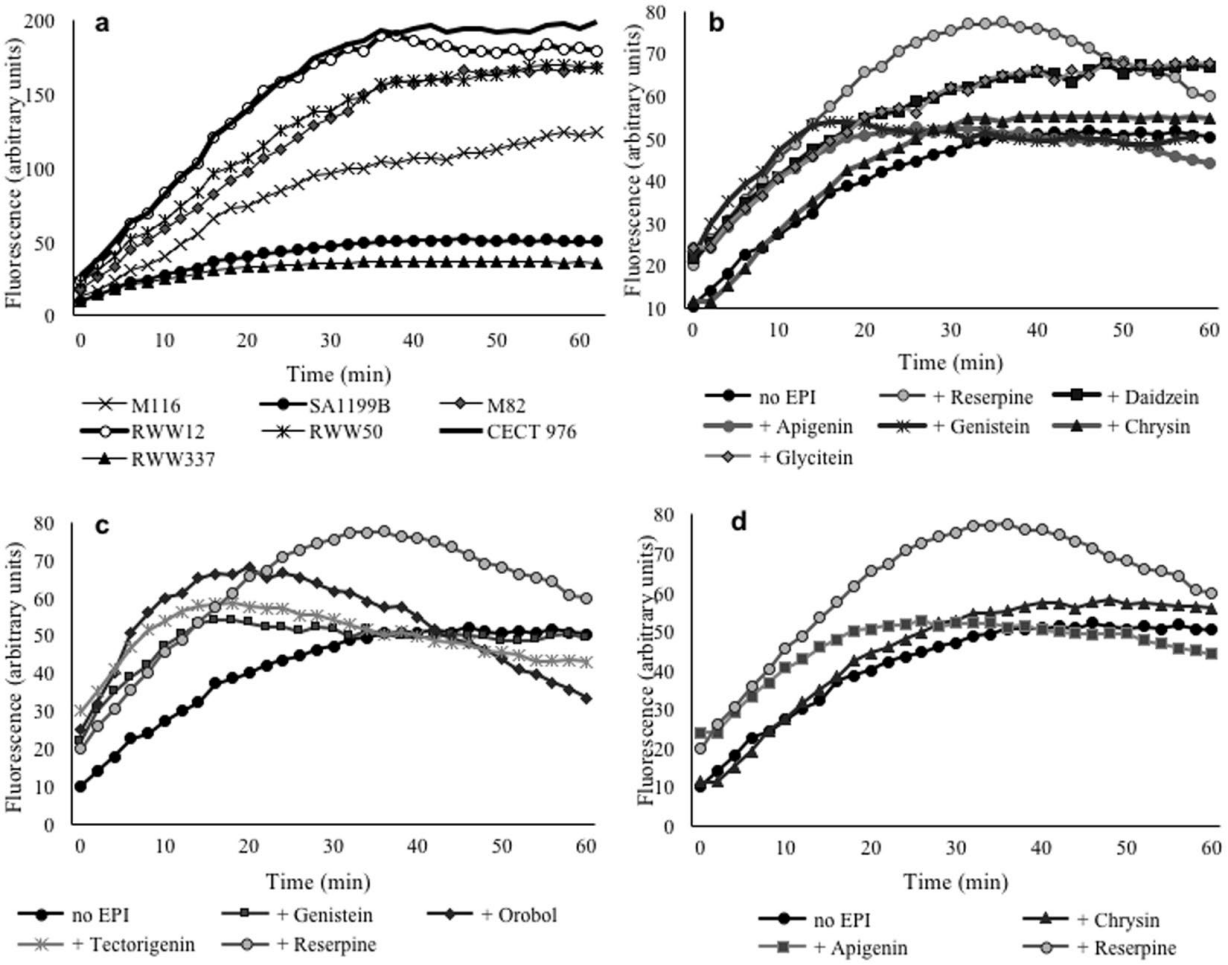
Effect of isoflavonoids on EtBr accumulation in *Staphylococcus aureus* SA1199B. In (**a**), fluorescence as a measure of EtBr accumulation is shown for all *Staphylococcus aureus* strains for 60 min at 37 °C; EtBr was applied at ½ MIC and fluorescence measurement obtained by fluorometric method; in (**b**), only the isoflavonoids (60 µg/mL) increasing the accumulation of EtBr in SA1199B cultures over control over time (*P* < 0.05) are represented; the changes in the accumulation of EtBr in SA1199B are also shown for genistein, tectorigenin (both at 60 µg/mL) and orobol (30 µg/mL) (**c**) and for the flavonoids apigenin and chrysin (**d**). Reserpine at 20 µg/mL was used as a positive control as efflux pump inhibitor (EPI). Mean values of least three independent experiments are shown.

The real-time setup and sensitivity of the fluorometric method allowed observation of the overtime accumulation of EtBr in *S*. *aureus* strains (Fig. 3a). As expected, EtBr accumulation was higher in susceptible strains, which present only a low basal expression of NorA and other efflux pumps. In Fig. 3(b–c), the effect of phytochemicals (at ½ MIC) on EtBr accumulation in the NorA overexpressing strain *S*. *aureus* SA1199B was analyzed. Reserpine (20 µg/mL), a recognized EPI, was used as a positive control. At a concentration of 60 µg/mL, all isoflavonoids were able to increase the accumulation of EtBr, except for orobol, which was already active at 30 µg/mL (data not shown for the other concentrations). As can be seen in Fig. 3b, isoflavonoids increased the accumulation of EtBr in SA1199B until approximately 40 min, after which the signal levels either stabilized or were slightly reduced again. Genistein, tectorigenin and orobol, and to a lesser degree apigenin, showed a different pattern (Fig. 3c), producing a significant increase in the accumulation of EtBr in the first 20–30 min (*P* < 0.05) after which values reverted to levels that were similar to (for genistein, *P* > 0.05) or lower than (for orobol and tectorigenin, *P* < 0.05) the control. Chrysin (found in *C*. *striatus*) had no effect on EtBr accumulation in SA1199B (Fig. 3d, *P* < 0.05). The positive effect of the aforementioned isoflavonoids on the accumulation of EtBr in SA1199B cells was also shown and confirmed by flow cytometry (Supplementary Experiment 2, Supplementary Fig. 3).

The activity of the isoflavonoids was also tested for strains M116, RWW337 and RWW50. Relative fluorescence (RF) values were calculated using the maximum fluorescence intensity (MFI) of each assay (Table 7). Independently of the differences in EtBr accumulation between the different MRSA strains, almost all the isoflavonoids increased the accumulation of EtBr in one or several strains, which indicates that they may target different proteins. Reserpine showed high values of RF for all strains. Among the isoflavonoids, biochanin A and tectorigenin caused the highest EtBr accumulation in MRSA strains. Calycosin and irigenin also showed high RF values (*P* < 0.05) for all MRSA strains, followed by genistein.

**Table 7:** Effect of isoflavonoids on the accumulation of ethidium bromide by fluorometry.

	SA1199B	RWW337	M116	RWW50
Reserpine	0.53 ± 0.19	0.25 ± 0.10	0.62 ± 0.14	0.75 ± 0.21
Daidzein	0.33 ± 0.10	0.05 ± 0.02	0.05 ± 0.09	−0.08 ± 0.06
Genistein	0.67 ± 0.19	0.20 ± 0.10	0.13 ± 0.11	0.12 ± 0.07
Tectorigenin	0.81 ± 0.12	0.19 ± 0.09	0.42 ± 0.07	0.47 ± 0.04
Glycitein	0.31 ± 0.10	0.06 ± 0.05	−0.02 ± 0.00	0.07 ± 0.05
Biochanin A	0.44 ± 0.05	0.63 ± 0.15	0.33 ± 0.08	0.47 ± 0.08
Calycosin	0.63 ± 0.11	0.19 ± 0.13	0.22 ± 0.03	0.34 ± 0.13
Irisflorentin	0.23 ± 0.03	0.05 ± 0.11	0.30 ± 0.05	0.02 ± 0.08
Irigenin	0.48 ± 0.15	0.15 ± 0.10	0.28 ± 0.04	0.34 ± 0.06
Orobol	0.76 ± 0.18	0.07 ± 0.04	0.08 ± 0.14	−0.13 ± 0.01
Apigenin	0.22 ± 0.05	0.01 ± 0.05	0.04 ± 0.03	0.05 ± 0.05
Chrysin	0.09 ± 0.07	−0.03 ± 0.02	−0.03 ± 0.10	0.01 ± 0.01

Relative fluorescence (RF) values were calculated for all isoflavonoids by fluorometric method in SA1199B and three MRSA strains. Reserpine was used as positive efflux pump inhibitor control. The flavonoids apigenin and chrysin found in *Cytisus striatus* leaves were included in the assay. The RF values are the averages and standard deviations of at least three independent assays.

## Discussion

It is known that plants can produce inhibitors as a protection against multidrug-resistant pathogens, thereby ensuring the efficient delivery of antimicrobial compounds. A good example of this is provided by Tegos *et al*.^5^, who found that *Berberis* plants, which produce a putative antimicrobial, berberine, also produce 5-methoxyhydnocarpin D (5-MHC-D) and pheophorbide A, which are MDR inhibitors and thus increase the accumulation of berberine in *S*. *aureus*. The role of phytochemistry in the search for *S*. *aureus* NorA inhibitors is significant, having led to the discovery of several chemically diverse plant-derived EPIs, including flavones, isoflavones, acylated glycosides, porphyrin phaeophorbide A and kaempferol rhamnoside^40^, among others^8^.

As a bioassay-guided fractionation does not generally detect synergistic and potentiating activities due to the risk that interacting molecules end up in different fractions, we used metabolomics as an alternative strategy. Successful implementation of a metabolomics approach requires the collection of many samples with different levels of the different plant metabolites to be able to reveal signals or peaks related to activity. These could be samples of different plants, or alternately, different extracts from the same plant and/or their fractions. An example on the use of fractions has been reported by Yuliana *et al*.^41^. In the present study, different extracts of the plant were obtained by mixing the different plant parts in different ratios and extracting them under different conditions. Multivariate data analysis of the NMR spectra and the results of the bioassays enabled the identification of signals that were correlated to high activity (Fig. 2). NMR-based metabolomics-guided fractionation was used to isolate the active compounds, which were identified as apigenin, chrysin, daidzein, genistein, 2′-hydroxygenistein and 3′-hydroxydaidzein. Apigenin showed no activity with concentrations below 120 µg/mL, in agreement with Basile *et al*.^42^, but not Sato *et al*.^43^. Similarly, Albert Dhayakaran *et al*.^44^ reported that MRSA strains were not inhibited by soy isoflavones, including genistein and daidzein (up to 100 μg/mL), and Morán *et al*.^45^ found no MIC for genistein up to 2 mg/mL against *S*. *aureus* CECT 59. Moreover, one antibacterial compound was identified in the *C*. *striatus* leaf extract – luteolin – with MICs of between 30 and 120 μg/mL against various *S*. *aureus* strains. Surprisingly, luteolin had lower MICs for RWW337 and M116-MRSA strains than for antibiotic-susceptible strains. Sato *et al*.^43^ also reported the antibacterial activity of luteolin with MICs of between 62.5 and 125 μg/ml against MRSA and MSSA strains. Among the isolated phenolics, apigenin and genistein clearly showed potentiation of the antibiotics against RWW337, M116 and RWW50 strains (Table 4). Both isoflavones, genistein and daidzein, showed ciprofloxacin potentiating activity against the resistant line SA1199B (8- and 4-fold increase, respectively). Similarly, genistein has been reported to reduce the MIC of norfloxacin against SA1199B four-fold and to have a moderate MDR efflux pump inhibitory effect^29^. Additionally, genistein showed antibiotic potentiation on the CECT 976 strain but neither genistein nor other phenolics showed the same result on the other susceptible strains M82 and RN6390. It is known that the results of antibacterial activity of phenolic compounds can be inconsistent, as reported by Cushnie and Lamb^14^, probably owing to variations in their susceptibility testing and differences in genetic determinants of the strains.

Isoflavones are thought to play a role in plant–microbial interactions as part of the defensive arsenal of the host plant, but neither this^26^ nor the exact relationship between chemical structure and activity is fully understood^24, 27, 46^. To further explore the possible synergistic activity, 22 isoflavonoids were tested for their antibiotic-potentiating effect on a number of resistant and susceptible *S*. *aureus* strains (Table 6).

Only three compounds revealed antimicrobial activity against all *S*. *aureus* strains: neobavaisoflavone (MIC of 20 µg/mL), corylifol A (MICs between 0.06 and 2.5 µg/mL), both of which have a lipophilic prenyl group attached to the B-ring, and orobol (MICs between 60 and 120 µg/mL). Besides genistein, biochanin A (a structurally similar isoflavonoid) and tectorigenin could potentiate ciprofloxacin and erythromycin (2- to 8-fold MIC reduction) against MRSA strains. A range of MIC values of 64–512 µg/mL against 12 *S*. *aureus* strains has been reported for biochanin A^47^. This compound also showed synergy with ciprofloxacin^47^. A MIC of 125 μg/mL against MRSA strains has been reported for tectorigenin^48^.

In our study, seven of the tested nonantimicrobial active isoflavonoids were able to potentiate ciprofloxacin activity (with 4- to 8-fold reductions of the MIC value) against the resistant SA1199B. In decreasing order of activity, these were genistein, biochanin A, tectorigenin, daidzein, calycosin, irigenin and irisflorentin (Table 6). Genistein and biochanin A were the most active of all the tested isoflavonoids, potentiating both ciprofloxacin and erythromycin against resistant *S*. *aureus* strains. A comparison of their structures suggests that the two hydroxyl groups in a meta position in the A-ring in these compounds are a key feature of this antibiotic potentiation. Orobol, which also has the same A-ring substitution but two *ortho*-hydroxyl groups in the B-ring, shows antibacterial activity. Tectorigenin, which differs from genistein by one additional methoxy group in C-6, also had significant potentiating activity. Daidzein, which lacks the 5-hydroxy group but is otherwise identical to genistein, is less active. It seems that synergistic properties parallel the estrogenic activity^49^. Furthermore, all compounds in which the C-7 hydroxyl group was glycosylated were inactive. The results reported by Hummelova *et al*.^25^ agree partially with our observations, since the isoflavonoids with the strongest *S*. *aureus* inhibitory effect were *ortho*-dihydroxyisoflavones. Other studies showed that 5,7-dihydroxylation of the flavonoid A-ring was important for anti-MRSA activity^25, 50, 51^ and monomethoxy B-ring derivatives were generally found to be more active (antibiotic synergy) than the disubstituted B-ring derivatives^29^.

The next question to answer was the possible mode of action of the synergistic activity. The uptake and efflux of the antibiotics are decisive for this activity. A higher accumulation of an antibiotic can be related to one of the following processes: the increase of nonselective diffusion, the presence of more or less selective transporters for uptake or inhibitors of the efflux transporters. The mechanism behind transport inhibition is not well understood. There is strong evidence that some EPIs, such as reserpine, can bind to several transporters, probably due to the low substrate specificity of these systems^52^. Nevertheless, it seems that the most active agents belong to the families of compounds possessing a conjugated system^40^. Ethidium bromide is a substrate for many efflux systems in various microorganisms, including *S*. *aureus* ^12^, and has been shown to be a particularly suitable probe for efflux studies, since it emits weak fluorescence in aqueous solution (external to the cell) and becomes strongly fluorescent when it binds to cellular components^53^. To measure the effect on the uptake and efflux, the accumulation of the highly fluorescent EtBr in the bacteria was measured in the presence of the isoflavonoids. Some isoflavonoids seemed to induce a fast EtBr accumulation, but over time the levels in the cell reverted to those of the controls, meaning that an equilibrium was reached after a certain time. The seven isoflavonoids that potentiated the activity of ciprofloxacin against SA1199B were those that most increased the accumulation of EtBr. No effect was observed in SA1199B when apigenin and chrysin (found in *C*. *striatus*) were combined with ciprofloxacin.

When studying efflux activity in MRSA clinical isolates instead of well-defined collection strains, the myriad of bacterial responses can complicate the clear interpretation of data. Bacterial response to ciprofloxacin is mediated, in most cases, not by one single efflux pump but by several (such as SdrM, MdeA, MepA, NorA, NorB or NorC), thereby hampering the perception of the role played by each individual pump in the overall efflux activity/resistance phenotype^54^. The same substrate can promote the expression of different efflux pump genes depending on its concentration and/or time of exposure. Also, isolates belonging to the same clonal type can have different levels of efflux activity and respond to the same agent through the activation of different efflux pumps^55^. The RWW337 strain showed a poor accumulation of EtBr, like SA1199B, followed by M116 strain, suggesting that, in contrast to the other strains, there is an overexpression of NorA or other related MDR efflux pumps in these strains. Nevertheless, biochanin A, tectorigenin and genistein improved EtBr accumulation as compared to the control in all MRSA strains (Table 7). Calycosin and irigenin had lower RFs, but still improved EtBr accumulation as compared to the control in all MRSA strains. These results suggested that an inhibition of efflux pumps, known to be involved in resistance mechanisms, or an effect on membrane permeability could contribute to the potentiation effect of the isoflavonoids. Cell membrane damage has been reported in connection with the isoflavonoids^48, 56^.

This study aimed to identify active antibiotic adjuvants from plants using a metabolomics approach. In previous studies, the plant *Cytisus striatus* showed interesting antimicrobial activity. By combining the metabolomics data with the activity of a number of different extracts prepared from the same plant, and by applying multivariate data analysis, it was possible to identify the signals that correlate with a potentiating effect on two antibiotics. These signals belonged to two types of plant secondary metabolites, flavonoids and isoflavonoids, the activities of which were partly known. The results prove that NMR-based metabolomics is a powerful tool for identifying synergistic activities, enabling the identification of the involved metabolites.

To investigate whether the activity observed with these particular compounds could be extended to other isoflavonoids, and if so, if there were some structural features related to the activity, 22 structurally different isoflavonoids were tested for their potentiating activity of ciprofloxacin and inhibition of the NorA efflux pump. This showed that the antimicrobial and antibiotic-potentiation properties of isoflavonoids are highly structure-dependent. All results are clearly promising and open the door to further research questions such as the structure-activity relationship of isoflavonoids for potentiating the activity of antibiotics, or more specifically in inhibiting efflux pumps, also possibly in other organisms. This might be interesting also for other potentiating effects on other pharmacological activities. Furthermore, given that the inhibition of NorA did not explain the potentiation observed for erythromycin, further work should be done to analyze the possible interference with other cellular efflux pumps. The concept of restoring and enhancing the therapeutic value of antimicrobials by employing EPIs is a formidable challenge. The development of combinations of fluoroquinolone antibiotics with a transporter inhibitor appears to be a feasible alternative to the discovery of “rare” antibiotics that are poorly recognized by multidrug transporters. Revealing the mechanisms plants may use to resist microbial infections, including molecules that affect the efflux of antimicrobials, may be promising for the discovery of compounds that potentiate and thus “recycle” antimicrobial drugs.

## Methods

### Plant material

*Cytisus striatus* (Hill) Rothm. was collected from Trás-os-Montes and Beira Transmontana (Portugal) in April and May of 2013 and identified by the botanical garden in Vila Real (Portugal). The leaves, flowers and twigs were harvested, separated, immediately frozen in liquid N_2_ and freeze-dried in order to avoid unwanted enzymatic reactions and stored at −20 °C until analysis.

### Preparation of plant samples and extracts

To study the activity of *C*. *striatus* leaves, flowers and twigs, 5 g of each of these plant materials were extracted with 50 mL of MeOH at 30 °C, while stirring at 150 rpm for 60 min. After filtration, the extracts were dried using a rotary evaporator and redissolved in 10 mL of methanol. This extract was partitioned with *n*-hexane (3 × 10 mL) to eliminate lipophilic compounds. The remaining methanolic phase was evaporated using a rotary evaporator and redissolved in H_2_O/MeOH (95:5). The resulting solution was extracted with 3 × 10 mL portions of ethyl acetate and the extracts were combined and evaporated with a rotary evaporator (EtOAc fraction). These extracts were analyzed by ^1^H-NMR and tested for both antibacterial and antibiotic-potentiating activities. The hexane and remnant H_2_O/MeOH fractions were also tested in order to ensure the absence of compounds of interest in these extracts (data not shown).

A multi-extraction strategy was performed for multivariate data analysis. To generate metabolic variation, leaves or flowers and twigs of *C*. *striatus* were mixed in different proportions (0, 25, 50, 70, 85, 100% (w _(leaf or flower)_/w _(twig)_). Approximately 1 g of each of these samples was extracted using different conditions of pressure and temperature with an E-916 speed extractor (Büchi, Flawil, Switzerland). The tested conditions were: temperature 30 or 90 °C, pressure 50 or 100 bar, solvent 50, 75 or 100% aqueous MeOH. For all conditions, the extraction was performed using two 4-minute cycles with a flow of 3 mL of solvent/min. The samples were labeled using a binary numbering system (*x*,*y*), where *x* specifies the extraction conditions and *y* the sample composition (Supplementary Table 2). In total, 54 different samples were extracted and vacuum-dried (Syncore, Büchi).

All the samples were redissolved in 10 mL of methanol and submitted to the fractionation scheme described previously. The ethyl acetate fractions were taken to dryness with a rotary evaporator, analyzed by ^1^H-NMR and tested for both antibacterial and antibiotic-potentiating activities.

### Bacterial strains

Seven *S*. *aureus* strains were used in this study. Further details are shown in Table 1. Prior to use, each strain, kept at −80 °C, was transferred onto Tryptic Soy Agar (TSA) with 5% sheep’s blood (BD, Breda, the Netherlands) plate, grown overnight and inoculated into MH broth at 37 °C with agitation (150 rpm). The antibacterial susceptibility of the strains was tested using a Vitek® II (bioMérieux); the antimicrobial susceptibility test was analyzed according to the CLSI guidelines^32, 57^.

### Antibiotics and phytochemical compounds

Ciprofloxacin and erythromycin were purchased from Sigma-Aldrich (St. Louis, USA) and stock solutions were prepared according to CLSI guidelines^57^. Three flavonoids, luteolin, apigenin and chrysin, and 22 isoflavonoids, biochanin A, calycosin, calycosin-7-*O*-β-D-glucoside, corylifol A, daidzein, daidzin, genistein, genistin, glycitein, glycitin, formonenetin, 3′-hydroxypuerarin, iridin, irigenin, irisflorentin, 3′-methoxypuerarin, neobavaisoflavone, ononin, orobol, puerarin, tectoridin and tectorigenin, were purchased from Biopurify, Chendu, China. Stock solutions were prepared in dimethyl sulfoxide (DMSO; Sigma-Aldrich, St. Louis, USA). Table 5 shows the chemical structures of all the flavonoids and isoflavonoids used in this study. Reserpine (Sigma-Aldrich), a recognized EPI^58, 59^, was used as positive control in EtBr accumulation assays and prepared in the same way.

### Antibacterial susceptibility testing

The MIC was determined using the microdilution broth method according to CLSI guidelines^32^. Bacteria (~10^6^ CFU/mL) were inoculated into MH broth and 200 μL/well were distributed in 96-well microtiter plates, along with twofold dilutions of the compounds to test. The MIC was defined as the lowest concentration of the compound that inhibited bacterial growth after 24 h of incubation at 37 °C. The bacterial growth was determined at 600 nm using a microplate reader (Spectramax M2e, Molecular Devices, Inc.). Three independent experiments were performed for each compound. The highest concentration of DMSO remaining after dilution (5%, v/v) caused no inhibition of bacterial growth.

### Antibiotic-potentiation testing – disk diffusion test

This method is most suitable for testing plant extracts, as it allows a better visualization and detection of the potentiating effects. The test was performed as described in previous studies^20, 35, 36^. Dried *C*. *striatus* extracts were prepared in DMSO. Each extract was added to previously autoclaved and cooled MH agar in the amount calculated to obtain the required final concentration. Then, 20 mL of medium were poured into 90 mm Petri dishes. The bacterial suspensions were adjusted to 0.5 McFarland standard and seeded over hardened MH agar Petri dishes using a sterilized cotton swab. Sterile blank discs (6 mm diameter; Oxoid) were placed on the agar plate seeded with the respective bacteria. A volume of 10 µL of each antibiotic prepared according to the CLSI guidelines (ciprofloxacin – 5 µg/disc; erythromycin – 15 µg/disc)^57^ was added to the blank discs. Antibiotic- and DMSO-impregnated discs on simple MH agar plates were used as positive and negative controls, respectively. The plates were incubated at 37 °C for 24 h. After incubation, each IZD was recorded and analyzed according to CLSI guidelines^57^. No inhibition zone was obtained with DMSO. All tests were performed in triplicate. The antibiotic-potentiating activity of *C*. *striatus* extracts was categorized, as described in previous studies^20, 35, 36^, into three classes according to the ratio of the increase of the IZD produced by the antibiotic in the presence of the plant extracts to that obtained with the antibiotic alone (on simple MH agar plates): 1 – indifferent (IZD combination – IZD antibiotic < 4 mm); 2 – additive (4 ≤ IZD combination – IZD antibiotic < 6 mm); and 3 – potentiation (IZD combination – IZD antibiotic ≥ 6 mm) of inhibition of growth of *S*. *aureus*.

### Antibiotic-potentiation testing – checkerboard

The checkerboard assay was performed according to CLSI guidelines^32^ as described in previous studies^20, 35, 36^. The MICs of ciprofloxacin and erythromycin were determined in the presence of increasing amounts of the tested compounds against *S*. *aureus* strains. Twofold serial dilutions in MH were carried out for the antibacterial compounds, yielding final concentrations ranging from 2 × MIC to 1/64 × MIC. Compounds with no detectable antibacterial activity were tested at several concentrations between 120 and 0.06 µg/mL. Drug combinations did not exceed 5% (v/v) of the volume used in each well (200 µL). Growth controls consisted in 5% (v/v) DMSO solutions and negative controls were prepared by adding fresh MH without bacteria to each drug combination. Incubation was performed for 24 h at 37 °C and measured spectrophotometrically at 600 nm. All MIC determinations were repeated at least in triplicate. Synergistic interactions between two drugs, one being an antibiotic (A) and the other being the adjuvant (B), were classified using the FIC index: FICI = FIC(A) + FIC(B), where FIC(A) is the ratio between the MIC of drug A in combination and the MIC of drug A alone, and FIC(B) is the ratio of the MIC of drug B in combination and the MIC of drug B alone^60^. A FICI value of ≤0.5 was interpreted as synergy. When the MIC B alone was not detected in the range of tested concentrations, double the highest concentration that was tested was used for FICI determination, and if the FICI value was ≤0.5, this interaction was assumed to be a potentiation^60^.

### NMR analysis

Each dried sample was mixed with 500 μL of CH_3_OH-*d*_*4*_, vortexed for 10 s, sonicated for 20 min at 42 kHz and then centrifuged at 13,000 rpm at room temperature for 5 min. The supernatant (300 μL) was transferred to a 3 mm micro-NMR tube and analyzed. ^1^H-NMR spectra were recorded at 25 °C on a 600 MHz Bruker DMX-600 spectrometer (Bruker, Karlsruhe, Germany) operating at a proton Larmor frequency of 600.13 MHz. Methanol-d_4_ was used as the internal lock. The ^1^H-NMR experimental parameters were the following: 128 scans requiring 10 min and 26 s acquisition time, 0.16 Hz/point, pulse width (PW) = 30° (11.3 μs) and relaxation delay (RD) = 1.5 s. FIDs were Fourier transformed with LB = 0.3 Hz. The resulting spectra were manually phased and baseline corrected, and calibrated to MeOH-*d*_4_ at 3.3 ppm, using TOPSPIN 3.2 software (Bruker BioSpin GmbH, Rheinstetten, Germany). 2D-NMR techniques (*J*-resolved, ^1^H-^1^H COSY, ^1^H-^13^C heteronuclear multiple-bond correlation (HMBC), and ^1^H-^13^C heteronuclear single quantum coherence (HSQC)), were performed when necessary as previously described^37, 61^.

### Isolation of the bioactive compounds

NMR-guided isolation was used to isolate the bioactive compounds from *C*. *striatus* leaves for their identification. For this, 55 g of leaves were extracted with 500 mL of MeOH and fractionated with ethyl acetate as described before in the section “Preparation of plant samples and extracts”. The resulting dry ethyl acetate fraction (1.2 g) was subjected to medium-pressure liquid chromatography (MPLC). Samples were introduced as solids using a pre-Elut column connected just before the main separation column (200 × 35 mm, i.d., Sepacore^®^ Silica 80 g, Büchi, Switzerland) and eluted using a gradient of chloroform (A) and methanol (B) (B:10%, 15 min; B:10–30%, 5 min; B: 30%, 20 min; B: 30–50%, 5 min; B:50%, 10 min) at a flow rate of 20 mL/min. The eluant was monitored at 220, 254, 280 and 365 nm. Sixty fractions were collected and combined into six fractions according to their TLC profile similarities. The TLC was performed on silica gel plates (Si60 HF_254_, Merk, Darmstadt, Germany) with CHCl_3_:MeOH:acetic acid (7.5:2.5:0.2). These six fractions were prepared for ^1^H-NMR analysis and for their antibacterial-potentiating analysis as described previously. One of the fractions (A3) proved to be active according to the disk diffusion method. This fraction (270 mg) was separated on a Sephadex LH-20 column with methanol at a flow of 3.5 mL/min and monitored at 220, 254, 280 and 365 nm. Sixty subfractions were collected and combined into six fractions (B1–B6) according to their TLC profile similarities. These were analyzed by ^1^H-NMR and tested for their antibacterial-potentiating capacity. Out of all these subfractions (B), two (B5 and B6) showed activity. B5 proved to have an antibiotic-potentiating activity with both ciprofloxacin and erythromycin and B6 had antibacterial activity against *S*. *aureus* strains. The B5 fraction (13.5 mg) was purified by semi-preparative reverse-phase high-performance liquid chromatography (RP-HPLC) (Luna C_18_ column; 250 × 10 mm, i.d., 5 μm, Phenomex^®^) and eluted with a gradient of 1% aqueous acetic acid (A) and methanol (B) (B: 1–42%, 30 minutes; B: t 42–100%, 10 minutes; B: 100%, 2 minutes). The detector was set at 270 nm. Six main fractions were obtained and analyzed by ^1^H-NMR analysis for the identification of metabolites, and 2D-NMR techniques were used when necessary. The B6 fraction (8.3 mg) contained only one compound that was identified as luteolin.

### EtBr accumulation assay by the fluorometric method

EtBr accumulation by the *S*. *aureus* strains on a real-time basis was detected using a fluorometric method in a 96-well plate fluorometer following modified published methods^12, 62^. The MIC of EtBr (prepared as a stock of 10 mg/mL) was determined for each strain according to CLSI guidelines as previously described. Briefly, *S*. *aureus* strains were grown in MH medium at 37 °C until the mid-log phase (OD_600_ of 0.6 to 0.7). Cultures were centrifuged at 13,000 rpm for 5 min, the supernatant was discarded and the pellet was washed in phosphate-buffered saline (PBS; pH 7.4). The OD_600_ was adjusted to 0.4 with PBS. Aliquots of 100 µl of bacterial suspension were transferred into the wells of a 96-well plate containing serial dilutions of EtBr at concentrations ranging from 80.00 to 0.06 µg/mL. In order to determine the effect of the isoflavonoids on the EtBr accumulation, EtBr (at ½ MIC) was applied in the absence of each isoflavonoid or with concentrations of 15, 30 and 60 µg/mL. Reserpine (20 μg/mL) was used as a positive control as it is a recognized EPI^39^. The negative control consisted of 1% (v/v) DMSO. Relative fluorescence was acquired every 60 s for 60 min at 37 °C in a Fluostar Optima (BMG Labtech, Ortenberg, Germany), using 530 nm and 590 nm as excitation and detection wavelengths, respectively. To be able to compare the EtBr accumulation experiments, the relative fluorescence (RF) was determined for each assay. The RF was calculated applying the equation RF_assay_ = (MFI_assay_ −FI_control_)/ FI_control_, where MFI_assay_ was the maximum fluorescence intensity (MFI) obtained in each 60 min assay and FI_control_ was the corresponding fluorescence intensity (FI) obtained with the DMSO control at the same time. High RF values indicated that cells accumulated more EtBr under the tested conditions than the control and vice versa for negative values. The experiments were repeated three times, and the RF values presented are the averages of the three independent assays.

### EtBr accumulation assay by flow cytometry

Flow cytometry facilitates the analysis of cells in suspension as they pass through a beam of light at a high rate in a fluid stream. Data acquisition and analysis were performed using a BD Accuri™ C6 flow cytometer (Breda, the Netherlands) equipped with three fluorescence detectors: FL1 (515 to 565 nm), FL2 (565 to 605 nm) and FL3 (>605 nm). The maximum absorption for EtBr is 518 nm and the maximum fluorescence emission is 605 nm. Preparation of *S*. *aureus* strains was performed as described in the fluorometric method and the strains were distributed in aliquots of 100 µL. The EtBr was added at ½ MIC and the isoflavonoids were added at the best concentration as defined with the previous method. Positive controls were performed with 20 µg/mL of reserpine and negative controls with 1% (v/v) DMSO. Following incubation at 25 °C for 60 minutes, the supernatant was removed and the pellet was resuspended in EtBr-free PBS, adjusting the OD_600_ to 0.3. Samples were taken for measuring fluorescence in the BD Accuri™ C6 flow cytometer. Data was collected for at least 10,000 events per sample. In this method, the final median fluorescence intensities were recorded and RF values were calculated as previously explained.

### Data analysis and statistics

The NMR spectra were bucketed using AMIX 3.9.12 (Bruker BioSpin GmbH, Rheinstetten, Germany). Buckets were obtained by integrating 0.04 ppm intervals and scaling the intensity of individual peaks to the total intensity recorded in the region *δ* 0.20–10.02. The regions of δ 4.85–4.95 and δ 3.20–3.40 were excluded from the analysis because of the residual signal of D_2_O and CD_3_OD, respectively. Orthogonal projection to latent structures (OPLS) based on Pareto scaling was performed with the SIMCA-P + software (v. 12.0, Umetrics, Umeå, Sweden). The outliers were not considered. The metabolites contributing to the separation were analyzed using an S-plot of the OPLS modeling.

For antibacterial-potentiating and EtBr accumulation tests, means and standard deviations were calculated and mean comparisons were made using the Student’s *t*-test (two-tailed based on a confidence level <0.05).

## Electronic supplementary material

Supplementary Data
