# Supplementary material for: Looking to nature for a new concept in antimicrobial treatments: isoflavonoids from Cytisus striatus as antibiotic adjuvants against MRSA
Source: Sci Rep. 2017 Jun 19;7:3777. doi: 10.1038/s41598-017-03716-7 (PMC5476642; doi:10.1038/s41598-017-03716-7)
Supplement: Supplementary file 1 — Supplementary Data [file 41598_2017_3716_MOESM1_ESM.doc]

**Supplementary Data**

**Looking to nature for a new concept in antimicrobial treatments: isoflavonoids from *Cytisus striatus* as antibiotic adjuvants against MRSA**

Ana Cristina Abreu^1,2^, Aline Coqueiro^1^, Andi R. Sultan^3^, Nicole Lemmens^3^, Hye Kyong Kim^1^, Robert Verpoorte^1^, Willem J.B. van Wamel^3^, Manuel Simões^2,*^, Young Hae Choi^1,*^

^1^Natural Products Laboratory, Institute of Biology, Leiden University, Leiden, the Netherlands.

^2^LEPABE, Department of Chemical Engineering, Faculty of Engineering, University of Porto, Rua Dr. Roberto Frias, s/n, 4200-465 Porto, Portugal.

^3^Department of Medical Microbiology and Infectious Diseases, Erasmus MC, Rotterdam, the Netherlands.

*Corresponding authors: Manuel Simões (mvs@fe.up.pt); Young Hae Choi (y.choi@chem.leidenuniv.nl)

**SUPPLEMENTARY EXPERIMENTS**

**(1) EtBr MIC determination.** The MIC of EtBr was first determined for each strain using the microdilution technique, resulting in 5 µg/mL for CECT 976, M82, RN6390, M116 and RWW50 and 40 µg/mL for SA1199B and RWW337.

**(2) Effect of isoflavonoids on accumulation of EtBr on SA1199B by flow cytometry.** The effect of the EPIs on the accumulation of EtBr on SA1199B cells was also shown and confirmed by flow cytometry through the acquisition of mean fluorescence intensities (FL3-A) after incubation of SA1199B for 60 min at 37 ºC with EtBr (1/2 MIC), reserpine (at 20 µg/mL) and each isoflavonoid (at the same concentrations previously described). EtBr accumulation was higher with genistein (similar to reserpine, *P* > 0.05) and biochanin A, followed by tectorigenin (Supplementary Fig. 3). Calycosin and irigenin were statistically different from the control (*P* < 0.05) but the results were lower. In this assay, the fluorescence values were only recorded after 60 min of incubation, and final fluorescence values are not necessarily the ones that best reflect EtBr accumulation for all isoflavonoids as referred in the manuscript.

**a**

**b**

**c**

**Supplementary Figure 1 |** **Representative ^1^H-NMR spectra of *C. striatus* extracts.** ^1^H-NMR spectra (600 MHz, in CD_3_OD, phenolic region) of (a) leaf, (b) flower and (c) twig methanolic extracts (EtOAc phases).

| 1. **Identification of the biomarkers correlated with antibiotic-potentiating activity** |
| --- |
|  |
| 1. **Isolation and identification of active compounds** |
|  |

**Supplementary Figure 2 | Elucidating diagram of the method developed in this study**. 1^st^ step (a) - Identification of the biomarkers correlated with antibiotic-potentiating activity; through a multivariate data analysis applied to ^1^H-NMR data; 2^nd^ (b) - Isolation and identification of active compounds.

**
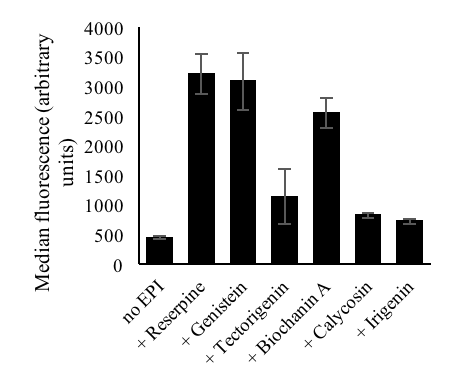
**

**b**

**c**

**d**

**a**

**Supplementary Figure 3 |** **Effect of isoflavonoids in EtBr accumulation in *S. aureus* SA1199B by flow cytometry.** (a) Median fluorescence values obtained by flow cytometry for *S. aureus* SA1199B after 60 min of exposure to EtBr (at 20 µg/mL) at 37 °C with reserpine (at 20 µg/mL) and isoflavonoids (at 60 µg/mL, except orobol at 30 µg/mL); only the EPI which fluorescence values statistically different from the control conditions (*P* < 0.05) are represented. Bars represent means and SD from at least three independent experiments. The effect of the EPIs on the accumulation of EtBr on SA1199B cells is similarly demonstrated and confirmed by flow cytometry with a histogram shift: (b) without EPI, (c) with genistein and (d) with biochanin A.

**Supplementary Table 1 | Antibiotic-potentiating activity of *C. striatus* leaf by checkerboard assay.** MICs (mg/mL) of *C. striatus* leaf methanolic extract (EtOAc fraction) against *S. aureus*, when applied alone (MIC_a_) and in combination (MIC_b_) with ciprofloxacin or erythromycin. Plant extract was tested in a range of concentrations from 0.06 to 1.0 mg/mL. Fold-reductions of antibiotic MICs are also represented (R) as well as FICI values. When FICI ≤ 0.5 (in bold), the effect is considered potentiation (P). ERY was not tested against SA1199B. The experiments were repeated three times, and the values presented are the averages of three independent assays

|  |  |  | **CIP** | | |  | **ERY** | | |
| --- | --- | --- | --- | --- | --- | --- | --- | --- | --- |
|  | **MIC_a_** |  | **MIC_b_** | **R** | **FICI** |  | **MIC_b_** | **R** | **FICI** |
| **CECT976** | > 1 |  | 0.25 | 4 | **≤ 0.38 (P)** |  | 0.25 | 4 | **≤ 0.38 (P)** |
| **M116** | > 1 |  | 0.25 | 4 | **≤ 0.38 (P)** |  | 0.5 | 4 | **≤ 0.5 (P)** |
| **RWW337** | > 1 |  | 0.25 | 4 | **≤ 0.38 (P)** |  | - | - | I |
| **RWW50** | > 1 |  | 0.125 | 4 | **≤ 0.31 (P)** |  | 0.5 | 2 | > 0.5 (I) |
| **M82** | > 1 |  | 0.5 | 2 | > 0.5 (I) |  | - | - | I |
| **RN6390** | > 1 |  | - | - | I |  | - | - | I |
| **SA1199B** | > 1 |  | 0.125 | 4 | **≤ 0.31 (P)** |  | n.p. | | |

- = no decrease or increase in the MIC was observed; n.p. not performed

**Supplementary Table 2 | Description of extraction conditions of *Cytisus striatus.*** Six extractions of different samples of *C. striatus* samples were performed, by mixing different percentages of leaves or flowers with the twigs for 1 g total sample. Nine runs were performed with variation of temperature (30 or 90 ºC), pressure (50 or 100 bar) and % MeOH (50-, 75- and 100% MeOH in water). A total of 54 samples were extracted. Identification of the samples was performed by numbering them as *(x,y)*, where *x* specifies the condition of the extraction and *y* the sample composition

|  |  |  | ***y* - Sample composition (%(w/w) leaf or flower in twig)** | | | | | | |
| --- | --- | --- | --- | --- | --- | --- | --- | --- | --- |
|  |  |  | **1**  **100 %** | **2**  **85%** | **3**  **70 %** | **4**  **50 %** | **5**  **25 %** | **6**  **0 %** | |
| ***x* - Conditions of extraction** | **1** | 100 % MeOH, 30 ºC, 50 bar | Leaf  (1,1) | Leaf  (1,2) | Leaf  (1,3) | Leaf  (1,4) | Leaf  (1,5) | | Leaf  (1,6) |
|  | **2** | 100 % MeOH, 90 ºC, 100 bar | Leaf  (2,1) | Leaf  (2,2) | Leaf  (2,3) | Leaf  (2,4) | Leaf  (2,5) | | Leaf  (2,6) |
|  | **3** | 75 % MeOH, 30 ºC, 50 bar | Leaf  (3,1) | Leaf  (3,2) | Leaf  (3,3) | Leaf  (3,4) | Leaf  (3,5) | | Leaf  (3,6) |
|  | **4** | 75 % MeOH, 90 ºC, 100 bar | Leaf  (4,1) | Leaf  (4,2) | Leaf  (4,3) | Leaf  (4,4) | Leaf  (4,5) | | Leaf  (4,6) |
|  | **5** | 50 % MeOH, 30 ºC, 50 bar | Leaf  (5,1) | Leaf  (5,2) | Leaf  (5,3) | Leaf  (5,4) | Leaf  (5,5) | | Leaf  (5,6) |
|  | **6** | 50 % MeOH, 90 ºC, 100 bar | Leaf  (6,1) | Leaf  (6,2) | Leaf  (6,3) | Leaf  (6,4) | Leaf  (6,5) | | Leaf  (6,6) |
|  | **7** | 100 % MeOH, 30 ºC, 50 bar | Flower  (7,1) | Flower  (7,2) | Flower  (7,3) | Flower  (7,4) | Flower  (7,5) | | Flower  (7,6) |
|  | **8** | 75 % MeOH, 30 ºC, 50 bar | Flower  (8,1) | Flower  (8,2) | Flower  (8,3) | Flower  (8,4) | Flower  (8,5) | | Flower  (8,6) |
|  | **9** | 50 % MeOH, 30 ºC, 50 bar | Flower  (9,1) | Flower  (9,2) | Flower  (9,3) | Flower  (9,4) | Flower  (9,5) | | Flower  (9,6) |
